# Highly sensitive integrated pressure sensor with horizontally oriented carbon nanotube network

**DOI:** 10.1186/1556-276X-9-49

**Published:** 2014-01-28

**Authors:** Muhammad Aniq Shazni Mohammad Haniff, Hing Wah Lee, Daniel Chia Sheng Bien, Aun Shih Teh, Ishak Abdul Azid

**Affiliations:** 1 Nanoelectronics Lab, MIMOS Berhad, Technology Park Malaysia, Kuala Lumpur 57000, Malaysia; 2 School of Mechanical Engineering, USM Engineering Campus, Universiti Sains Malaysia, Nibong Tebal, Pulau Pinang 14300, Malaysia

**Keywords:** Carbon nanotube, Horizontally oriented, Flexible substrate, Pressure sensor, Sensitivity

## Abstract

This paper presents a functionalized, horizontally oriented carbon nanotube network as a sensing element to enhance the sensitivity of a pressure sensor. The synthesis of horizontally oriented nanotubes from the AuFe catalyst and their deposition onto a mechanically flexible substrate via transfer printing are studied. Nanotube formation on thermally oxidized Si (100) substrates via plasma-enhanced chemical vapor deposition controls the nanotube coverage and orientation on the flexible substrate. These nanotubes can be simply transferred to the flexible substrate without changing their physical structure. When tested under a pressure range of 0 to 50 kPa, the performance of the fabricated pressure sensor reaches as high as approximately 1.68%/kPa, which indicates high sensitivity to a small change of pressure. Such sensitivity may be induced by the slight contact in isolated nanotubes. This nanotube formation, in turn, enhances the modification of the contact and tunneling distance of the nanotubes upon the deformation of the network. Therefore, the horizontally oriented carbon nanotube network has great potential as a sensing element for future transparent sensors.

## Background

Carbon nanotubes (CNTs) are nanostructured materials used in the production of microelectromechanical sensors because of their outstanding electronic, mechanical, and electromechanical properties [1-3]. CNTs have gauge factors that exceed 2,900, which is an order or a magnitude higher than those of state-of-the-art silicon-based resistors [4]. The excellent strain of CNTs produces a highly piezoresistive network, which benefits pressure sensors and microscale/nanoscale strains with fine resolution. Many studies have examined the fabrication of highly sensitive pressure sensors by depositing piezoresistive CNTs onto the fixed silicon substrate [5-8], in which single-walled and multi-walled carbon nanotubes (SWNTs and MWCNTs, respectively) are utilized as active sensing elements [9,10].

Recently, flexible electronic devices attract considerable research attention because of their flexibility and transparency [11]. However, the deposition of highly uniform CNTs onto the flexible substrate is hindered by numerous challenges. Two techniques, namely solution deposition and transfer printing method, are proposed for such deposition [12,13]. Transfer-printed, chemical vapor deposition (CVD)-grown CNTs often outperform solution-deposited CNTs because of their highly aligned formation. Through the CVD method, the size, shape, and area density of CNTs are determined by the chemical composition, plasma, and geometrical features of the catalyst [14-17]. The sensitivity of as-grown CNTs on the application of load is determined by their formation. Therefore, the density and growth formation of as-grown CNTs must be optimized to enhance their pressure sensitivity.

In this paper, the incorporated horizontally oriented MWCNT network on a flexible substrate as a sensing element is presented for the purpose of enhancing sensitivity of pressure sensors in low-pressure applications. The controlled growth formation of this network is determined using an AuFe bilayer as a catalyst. The transfer of resultant nanotubes on the flexible substrate is examined, and their surface morphology and electrical properties are compared to those of the as-grown nanotubes.

## Methods

**Figure 1:**
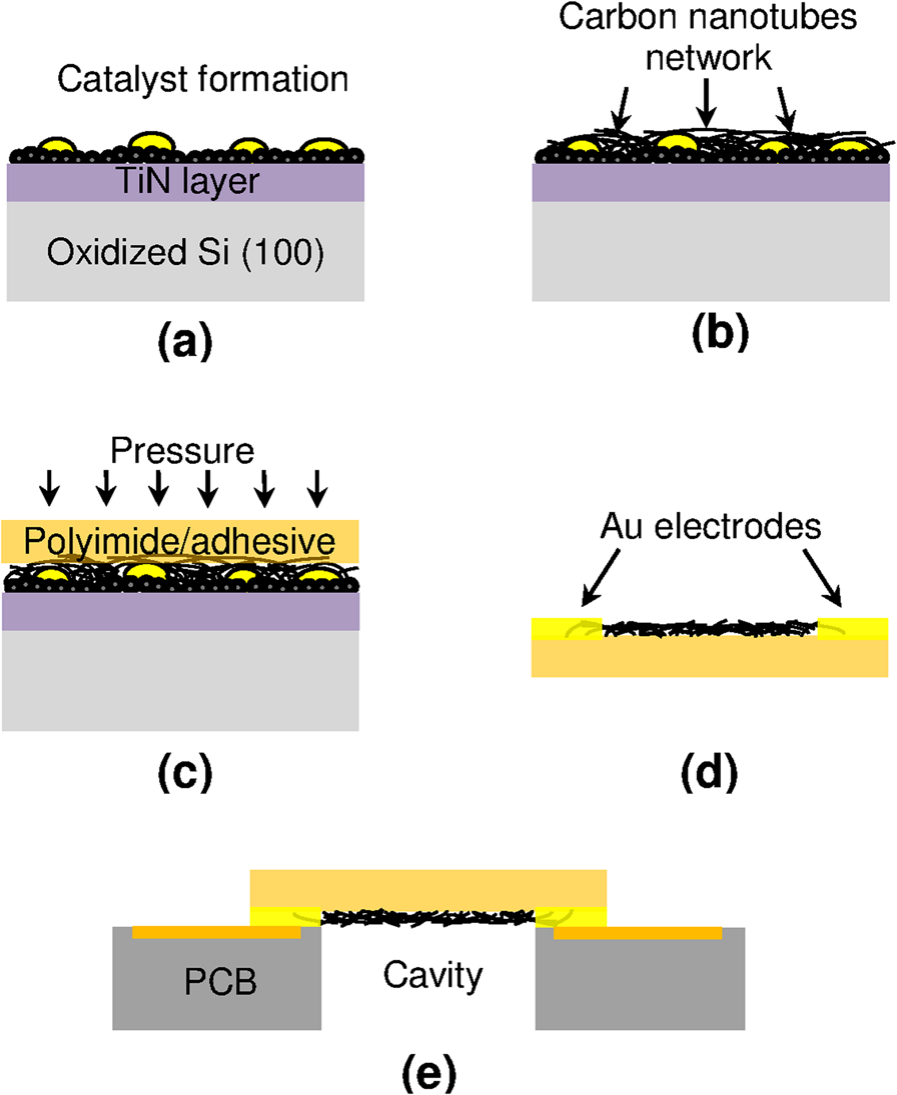
**Schematic of incorporation of MWCNTs on the flexible substrate. (a)** Annealing of catalyst thin films, **(b)** growing of carbon nanotubes, **(c)** transfer printing of carbon nanotubes on the polyimide adhesive substrate, **(d)** deposition of electrodes, and **(e)** integration of PCB.

Figure 1 shows the fabrication process of horizontally oriented MWCNTs on the flexible pressure sensor. Before the nanotubes were grown in a plasma-enhanced CVD, a catalytic thin AuFe film (10 nm) with a diffusion barrier layer of TiN (10 nm) and a thickness of 5 nm was deposited on a thermally oxidized Si (100) substrate via radio-frequency magnetron sputtering at approximately 10^-3^ mbar chamber pressure. An H_2_ plasma treatment with a 100-sccm flow rate and a 200-W plasma power was annealed on the as-deposited catalyst for 10 min at 700°C to form a seed layer with small nanoparticles. The nanotubes were grown on the AuFe seed layer with a 50-sccm acetylene (C_2_H_2_) flow rate at a 1,000-mTorr chamber pressure for 30 min. The resultant nanotube network from the Si (100) substrate was directly transferred to a 150-μm-thick polyimide adhesive substrate by sticking the network to the substrate with minimal pressure. Afterwards, the polyimide adhesive substrate with the as-transferred nanotube network was carefully peeled from the Si (100) substrate. The as-transferred nanotube network had a 6?×?1 mm^2^ effective area. A thick layer of 250-nm Au electrodes was sputtered on both ends of the as-transferred nanotube network to develop a flexible pressure sensor. These electrodes were then patterned by applying a transparent hard mask. After the electrodes were deposited, the as-transferred nanotube network was integrated onto a printed circuit board (PCB) with a cavity diameter of 6 mm as the pressure sensor based on the circular membrane. The catalyst formation, nanotube morphology, and electrical properties of both the as-grown and as-transferred nanotube networks were characterized using a JEOL (Tokyo, Japan) field emission scanning electron microscope with a 10-kV electron energy and a Hall effect measurement system. For the experimental setup, the fabricated pressure sensor was sealed and clamped completely on a test jig by epoxy bonding to prevent gas leakage. The differential applied pressure up to 50 kPa from N_2_ gas supply system to cavity was controlled and monitored by an ultralow pressure regulator and a commercial pressure sensor. Both the diaphragm and carbon nanotube network experience deformation under applied pressure. The resistance changes as a function of applied pressure were recorded by using a digital multimeter at room temperature.

## Results and discussion

**Figure 2:**
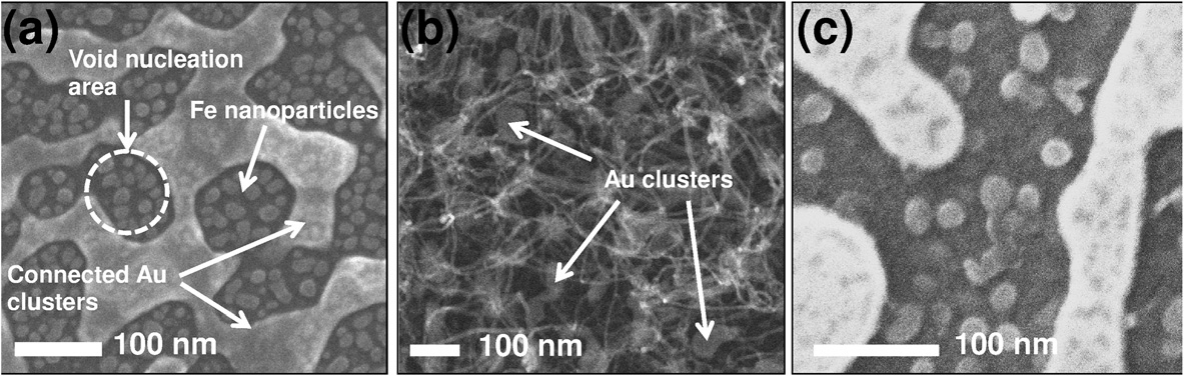
**Formation of catalyst and characteristics of the resultant MWCNTs on TiN/thermally oxidized Si (100). (a)** SEM image of the AuFe catalyst after annealing, **(b)** growth of the resultant MWCNTs for 30 min, and **(c)** SEM image of the peeled surface of MWCNTs.

Figure 2a shows the formation of AuFe nanoparticles distributed after a 10-min annealing process. The size distribution of the Au clusters that were connected with the small isolated Fe nanoparticles ranged from 16.9 to 200 nm. The agglomeration of Au and Fe films slightly differed because of the variation lattice mismatch in the thermal coefficient. The Fe nanoparticles were trapped in the void nucleation area between the Au clusters, which were produced by the grooving of the grain boundary. Figure 2b shows the MWCNTs grown on the AuFe catalyst. A horizontally oriented MWCNT network was formed with the remaining Au clusters on the substrate, which indicated the absence of growth on these clusters. In this case, the Au clusters formed a passivation layer to suppress nanotube growth, whose growth rate primarily depended on the availability of Fe nanoparticles. From least density of Fe nanoparticles, the nanotube growth occurred at a much lower rate of 0.02 μm/min with horizontally lying MWCNTs on the substrate as a result of weak attraction forces of the van der Waals among the neighboring nanotubes. The ends of the nanotubes were linked and overlapped among the neighboring tubes, hence forming a netlike structure. The growth rate of the CNT-based Fe catalyst was approximately 900 times lower than that reported by Moulton et al. [18], which resulted in a low-density formation.

Figure 2c shows the peeled surface of the nanotubes grown on the AuFe catalyst. A base growth mechanism was evidenced by the presence of Fe nanoparticles on the substrate, which was similar to the findings of Bower et al. [19]. Table 1 summarizes the characteristics of the catalyst nanoparticles and the growth of the resultant nanotube. The distribution of the resultant nanotubes was smaller than their catalyst in terms of diameter. This result could be attributed to the restriction of nanotube growth on the Fe nanoparticles, a growth caused by the strong interface reaction between the Fe nanoparticles and the TiN layer.

**Table 1:** Characteristics of the catalyst nanoparticles and the growth of the resultant nanotubes

**Type of catalyst/CNTs**	**Formation**	**Range of size/diameter (nm)**	**Density (×10**^**10**^**/cm**^**2**^**)**	**RMS (nm)**	**Growth rate (μm/min)**
AuFe catalyst	Connected clusters with small nanoparticles	16.9 to 200	9.07	4.81	-
MWCNTs	Horizontally oriented	7.0 to 9.0	22.31	5.36	0.02

**Figure 3:**
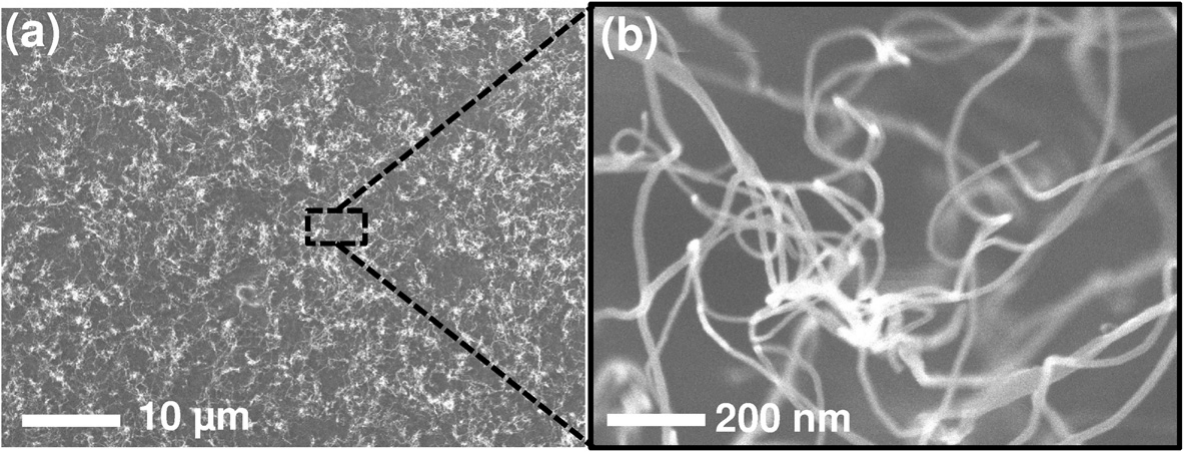
**SEM images of the as-transferred MWCNTs on the flexible substrate. (a)** Horizontally oriented MWCNT network and **(b)** close-up view from the top image.

Figure 3 shows the SEM images of the as-transferred horizontally oriented MWCNT network on the flexible substrate. Most of these CNTs retained their shapes on the flexible substrate without any significant changes in diameter and length, achieving a 90% yield rate. The adhesion between the adhesive underlayer and the flexible substrate was assumed to be much stronger than that between the as-grown horizontally oriented nanotubes and the TiN layer/thermally oxidized Si (100) substrate. Zhu et al. claimed that the poor adhesion between the high-temperature-synthesized nanotubes and the supporting substrate resulted from the weak van der Waals [20]. With regard to electrical properties, the sheet resistance of the as-grown and as-transferred MWCNTs was 5.3 and 7.7 kΩ/sq, respectively. The higher sheet resistance of the as-transferred MWCNTs was attributed to the scattering of electrons in the nanotube network on the flexible substrate. It is also worth to point out that the transport of electrons in the as-grown MWCNT network was enhanced by the conductive channels of the connected Au clusters with lower sheet resistance.

**Figure 4:**
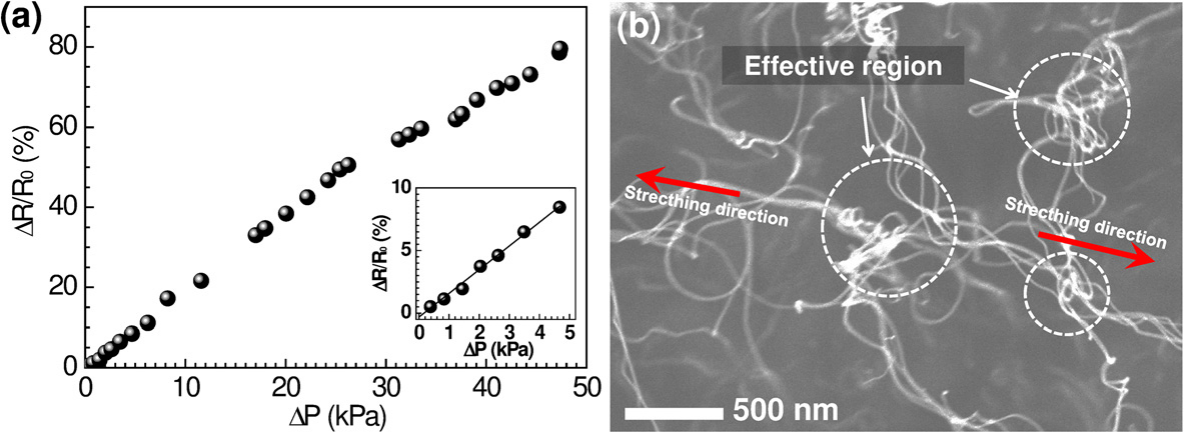
**Pressure-sensing performance of the horizontally oriented MWCNTs. (a)** Relative change in resistance after the application of pressure. The inset shows a plot of resistance changes, which range from a small scale of applied pressure to 5 kPa. The initial resistance *R*_0_ is measured at 150 kΩ. **(b)** Structure of the nanotubes during stretching.

Figure 4a shows the relative change in resistance of the horizontally oriented MWCNT network as a function of applied pressure. The performance or sensitivity of the pressure sensor was computed as *S*?=?(Δ*R*/*R*_0_)?×?100%/Δ*P* and expressed as percentage per kilopascal (%/kPa). An increased relative change in resistance was observed as the applied pressure was increased. The sensitivity of the horizontally oriented MWCNT network pressure sensors was calculated at approximately 1.68%/kPa, which reflected their high sensitivity to a small pressure change. Compared to other CNT-based pressure sensors, the sensitivities of the proposed pressure sensor was approximately 2, 3.5, 27, and 17 times higher than those reported by Su et al. [21] (carbon microcoils), Lim et al. [22] (CNT thin film), Park et al. [8] (carbon fiber), and Bsoul et al. [10] (vertically aligned CNTs forest), respectively. Such outperformance emphasizes the role of nanotube formation in enhancing sensitivity under applied pressure. It is expected that most of the resistance in the nanotube network is largely associated with the contact and tunneling resistances between adjacent nanotubes. A wide tunneling distance was observed between the isolated nanotubes in the larger end connections of the horizontally oriented MWCNT network, which reduced the contact area due to the low-density formation.

After applying pressure onto the membrane, the MWCNTs that were stretched via mechanical deformation likely modified the physical structure of the nanotubes in the effective region, which resulted in a loss of contact and an increase in the tunneling distance among the nanotubes as shown in Figure 4b. The contact area and the tunneling distance per nanotube were enhanced during the stretching because of the large portion of isolated nanotubes and the weak van der Waals forces among the nanotubes. Therefore, the electrons that hop between the adjacent nanotubes were reduced, limiting the transport of electrons in a region with lesser contact. Therefore, larger resistance changes resulted from the increased tunneling and contact resistance.

## Conclusions

This study used a flexible substrate to incorporate a highly sensitive horizontally oriented MWCNT network in pressure sensing performance. A horizontally oriented MWCNT network with low density was grown on an AuFe catalyst. The nanotube network was successfully transferred from the silicon-based substrate to a flexible substrate with 90% yield rate. Both the as-grown and as-transferred nanotubes were characterized to examine the variations in their morphologies and electrical properties. The fabricated pressure sensor showed a great potential in sensing a small change of pressure with a sensitivity of approximately 1.68%/kPa. A larger portion of isolated nanotubes could enhance the modifications of the contact area and tunneling distance per nanotube, which limited the transport and hopping of electrons due to the loss of contact among the nanotubes. Such modifications eventually increased the resistance changes and pressure sensitivity of the network.

## Competing interests

The authors declare that they have no competing interests.

## Authors' contributions

MASMH designed and conducted all experiments and characterizations and drafted the manuscript. HWL, DCSB, and AST conceived the research flow and helped in the technical support for experiments and in drafting the manuscript. IAA supported in the verification and interpretation of results. All authors read and approved the final manuscript.
